# Determining gene expression on a single pair of microarrays

**DOI:** 10.1186/1471-2105-9-489

**Published:** 2008-11-21

**Authors:** Robert W Reid, Anthony A Fodor

**Affiliations:** 1 Department of Bioinformatics and Genomics, The University of North Carolina at Charlotte, 9201 University City Boulevard, Charlotte, NC 28223, USA

## Abstract

**Background:**

In microarray experiments the numbers of replicates are often limited due to factors such as cost, availability of sample or poor hybridization. There are currently few choices for the analysis of a pair of microarrays where N = 1 in each condition. In this paper, we demonstrate the effectiveness of a new algorithm called PINC (PINC is Not Cyber-T) that can analyze Affymetrix microarray experiments.

**Results:**

PINC treats each pair of probes within a probeset as an independent measure of gene expression using the Bayesian framework of the Cyber-T algorithm and then assigns a corrected p-value for each gene comparison.

The p-values generated by PINC accurately control False Discovery rate on Affymetrix control data sets, but are small enough that family-wise error rates (such as the Holm's step down method) can be used as a conservative alternative to false discovery rate with little loss of sensitivity on control data sets.

**Conclusion:**

PINC outperforms previously published methods for determining differentially expressed genes when comparing Affymetrix microarrays with N = 1 in each condition. When applied to biological samples, PINC can be used to assess the degree of variability observed among biological replicates in addition to analyzing isolated pairs of microarrays.

## Background

Numerous strategies have been devised to come up with rigorous methods for detecting differentially expressed genes in sets of microarray data (for review see[1]). The majority of microarray analytical methods require N = 3 in each condition in order to perform statistical measures. Due to the expense of microarrays, experimental imperfections such as poor hybridization and limited quantities of available biological sample source, it is not always possible to obtain the required sample sizes.

On an Affymetrix expression array, such as the HG-U133A GeneChip, each gene is represented on the array by a number of separate 25 mer probes that correspond to a part of the gene sequence. Many popular statistical methods including MAS5[2], RMA[3] and GCRMA[4] summarize probes into a single value for the entire probeset before performing statistical inference. In contrast, probe-level modeling has been used by the software packages affyPLM[5] for quality-control purposes. There are also a number of statistical models that directly utilize probe information in statistical inference including Logit-T [6], Fisher's combined p-value [7], gMOS [8], and multi-mgMOS [9]. Despite performing inference on probes, rather than on probesets, these methods still require multiple experiments (N ≥ 3) in each condition.

In this paper, we explore the idea that it should in principle be possible to use the high number of probes in each probe set to substitute for repeat experiments. That is, instead of using repeated chips to estimate the variance for statistical inference, can we exploit the existence of multiple probes per probe set to estimate the variance? Previously, Hein and Richardson have used a Bayesian hierarchical model to estimate expression levels from probe level data allowing for analysis with N = 1 in each condition[10]. In their algorithm, called BGX[11], inference is performed at each stage of analysis (background correction, gene expression estimation and differential expression)[10]. The BGX algorithm outperformed[10] existing probe level metrics such as the Wilcoxon test statistic and another Bayesian algorithm EBarrays[12]. Because the BGX algorithm requiring a Markov chain Monte Carlo (MCMC) model for each stage of microarray analysis it is a very computationally demanding technique. As an alternative, we introduce an algorithm called PINC (PINC is not Cyber-T) based on the Cyber-T algorithm first described by Bali and Long[13] and a method we recently described for generating accurate p-values[14]. We show that PINC has attractive characteristics when compared to Cyber-T, BGX and other methods of performing inference on Affymetrix microarrays at low sample sizes.

## Results and discussion

### The Performance of Test Statistics in ranking genes on a control data set at N = 1

On the Affymetrix Latin Square HG-133A dataset, there are 11 probes per probeset. Given 11 independent measures in two samples, there are a variety of statistical tests available to evaluate the null hypothesis for each gene that the expression observed in each sample is identical. These include the Standard-T test, a paired t test (which is equivalent to a two way ANOVA in which the independent variables are probe and sample) and the Wilcoxon test[15] (a non-parametric equivalent to a paired-T test). In addition to these canonical statistical tests, there are variants of the t-test specifically designed for microarrays. These include the paired and unpaired Cyber-T tests [13] in which the variance for each gene is an estimate based on an average of the canonical variance for that gene and a background variance of other genes with similar intensities on each array (see methods).

**Figure 1:**
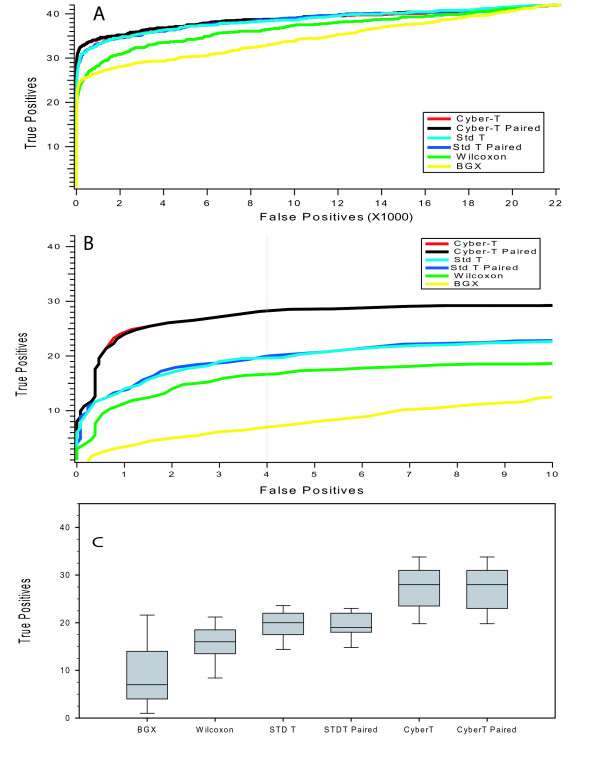
**Average ROC curves for 13 Latin Square experiments**. The performance of ranking true and false positives for pairs of N = 1 experiments are depicted. The first experiment from 13 2× Latin Square experiments was selected for analysis. For each of the 13 comparisons, an ROC curve was generated. Shown is the average of all 13 ROC curves. Figure 1A shows the full-scale performance for all false positives. Figure 1B is a zoomed in view of 1A with the x and y-axes zoomed to show detail of restrictive cutoffs with few false positives. Figure 1C is a box plot of the number of TP detected at an arbitrary cut off level of 4 FP (vertical dashed line in 1B).

We applied these different statistical measures to the Affymetrix Latin Square HG-133A dataset, which consists of 14 conditions of 3 replicates each. Each condition has 42 known genes spiked in at different concentrations that are true positives while the remaining 22,181 probe sets on the chip are true negatives. We examined the first replicate from each of the 14 experiments and compared experiments where there is a 2-fold change in spiked in concentration resulting in 13 separate comparisons (Exp 1 vs. Exp 2, Exp 2 vs. Exp 3, etc.). Applying the test statistics to these datasets yields for each statistic a gene list ranked according to the calculated scores. For each of these 13 comparisons, we can generate ROC curves of the number of true positives versus false positives at each possible cutoff for these gene lists with N = 1 in each condition. Figure 1A shows the average of these 13 ROC curves in which the x-axis displays all 22,181 true negatives. At this scale, it is immediately obvious that the BGX and Wilcoxon tests underperform the other statistics while the paired and unpaired Cyber-T tests perform the best.

While the data in Figure 1A give a broad overview of how the algorithms perform, the scale of the x-axis does not represent a biologically useful signal. For example, at a false positive rate of 0.05 a gene list for the HG-133A microarray would have over 1,000 false positives. Clearly such a gene list is not that useful. To better explore a more biologically relevant cutoff, in which a gene list consists of mostly true positives, Figure 1B shows the same data as in Figure 1A, but with the x-axis scaled to show only gene lists that include a small number of false positives. Figure 1C shows the number of true positives captured at a cutoff of N = 4 false positives (Figure 1B dashed vertical line) for all 13 comparisons. At this more stringent cutoff the paired and unpaired Cyber-T tests clearly outperform the other statistics.

### The Performance of Test Statistics in Providing Accurate p-Values for Inference

ROC curves rank all of the genes in an experiment but generating a gene list in a "real" experiment also requires choosing a cutoff point. That is, it is not enough to rank genes into an ordered list, one must know how many genes to consider significant from the list. Each test statistic generates a score for each gene and we wish to determine the threshold score above which genes are considered to be significantly differentially expressed. This has proven to be a challenging problem. In the microarray literature it is generally accepted that family-wise error rates, such as Bonferroni correction, are too conservative in an effort to prevent type-I errors thereby producing an abundance of type-II error [16,17]. The use of false discovery rates (FDR) has become a popular alternative for controlling error rates (for a review, see [16]).

In this study, we evaluated the performance of different test statistics using the Benjamini and Hochberg (hereafter BH)[18] and Benjamini and Yekutieli (hereafter BY)[19] FDR cutoff levels (see methods), as well as the Holm's step down method, a more conservative family wise error rate correction algorithm ([20] and see methods). For the FDR algorithms, we set the cutoff level at 10%, i.e., we are willing to accept that 10% of the genes considered to be significant will be false positives. For the Holm's step down FWER, we set a cutoff level of 0.05 divided by N (22,223) for the highest scoring gene pair. Then for each subsequent gene, the cutoff is recalculated as 0.05 divided by the number of remaining genes.

**Figure 2:**
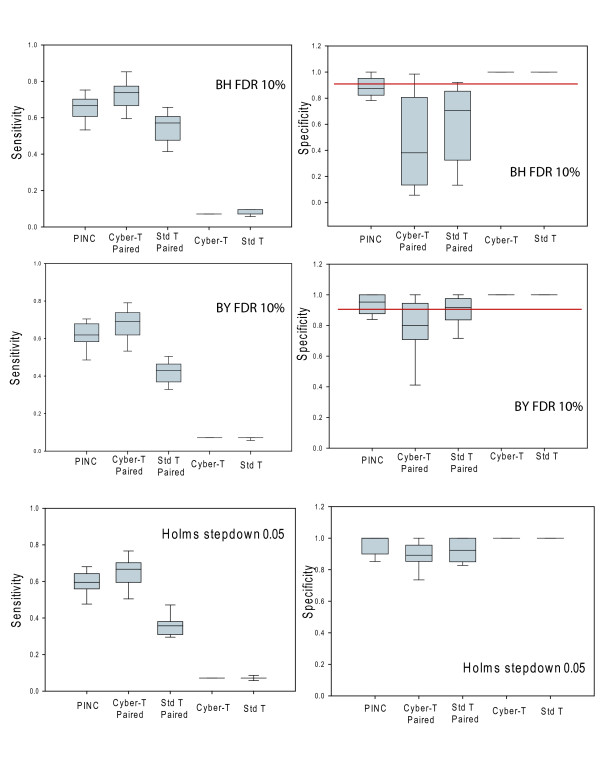
**Sensitivity and specificity for different algorithms applied to the 13 N = 1 2× Comparisons from the Latin Square dataset**. Left panels are sensitivity scores at different p-value cut off levels and panels on the right are specificity scores. The red lines in the top 2 right panels represent the predicted FDR cutoff value at 10%.

Figure 2 shows the sensitivity and specificity for the 13 N = 1 comparisons we performed on the Latin Square dataset for p-values produced by various methods under a 10% BH and BY FDR cutoff and a 0.05 Holmes step down cutoff. We define sensitivity as the number of true positives recovered at each threshold divided by the total number of true positives in the Latin Square data set. We define specificity as the number of true positives recovered at each threshold divided by the total number of genes above the threshold cutoff. An algorithm that generates p-values that are too large would be inappropriately conservative and not consider enough genes significantly differentially expressed. Such an algorithm would yield results with poor sensitivity but high specificity. Under all 3 cutoff schemes, this describes the Wilcoxon non-parametric test, which failed to detect any genes about our cutoff threshold (sensitivity = 0) and is therefore not included in Figure 2 or in further analyses. Because of the poor performance (Figure 1) and high computational cost of the BGX algorithm, it too was not included in this analysis. Of the remaining algorithms, we see that the unpaired Cyber-T and paired and unpaired Standard-T tests also produce p-values that are too large as they yield nearly perfect specificity but poor sensitivity. By contrast, an algorithm that produces p-values that are too small will yield results with high sensitivity but poor specificity. We see that under the BH and BY FDR schemes, this describes the paired Cyber-T test. With a 10% FDR threshold, we would expect a specificity of 0.9 (red lines in Figure 2). While the paired Cyber-T test is able to detect a large number of the true positives (highest sensitivity), it also incorrectly detects numerous false positives, resulting in a specificity measure well below the expected level of 0.9. We can say therefore that the paired Cyber-T test has failed to control false discovery rate under BH and BY FDR.

We have previously shown that, when applied at the probeset level, p-values produced by canonical statistics and the unpaired Cyber-T test are not very accurate on control Affymetrix datasets [14]. We proposed as a simple alternative, a method that assumes that all the background values on a microarray form a single distribution ([14] and see methods). We here propose a new algorithm PINC (PINC Is Not Cyber-T), which is the paired Cyber-T test performed at the probe level in which the p-values provided by the Cyber-T test are replaced with p-values generated by this assumption of a single background distribution. Applying the PINC algorithm yields a list in which the rank order is identical to the paired Cyber-T test (and therefore would have the same ROC profile in Figure 1) but the p-values differ. In Figure 2, we see that p-values generated by PINC do a better job of controlling FDR under both BH and BY FDR; the sensitivity of PINC in nearly as good as the sensitivity shown by Cyber-T paired, but the specificity is much closer to the expected level of 0.9. Indeed, no matter which of the three cutoff schemes we used to determine the threshold p-value of significance, the PINC algorithm nicely balanced sensitivity and specificity picking up a substantial fraction of true positives with a minimal number of false positives (Figure 2). All other algorithms perform poorly on either sensitivity and specificity suggesting that p-values calculated with these algorithms are either inappropriately large or inappropriately small. We conclude that when compared to other algorithms, the p-values produced by the PINC algorithm lead to inference that is less susceptible to bias introduced by the method of determining the threshold cutoff. That is, we argue that the p-values produced by PINC are more robust than p-values produced by the Cyber-T software or by canonical statistical tests.

### Consistency in technical and biological replicates

Our results suggest that, at least on the technical replicates of the Latin Square experiment, the PINC statistic produces p-values that allow for correct inference in discriminating true and false positives. Because the p-values generated in 1 vs. 1 comparisons do not involve biological replicates, they cannot be used to evaluate biological variability; that is, they do not indicate the reliability of the observed difference in gene expression relative to biological noise across individuals. Rather, the p-values reflect the magnitude of the differences between the samples relative to technical variability that arise from hybridization noise, optical noise, differences in RNA degradation between the samples, artifacts that arise from probe selection and so forth. For the tightly controlled datasets such as the Latin Square dataset, the performance of the PINC algorithm at assigning p-values reflecting these sources of noise at N = 1 is clearly acceptable (Figure 2). However, what happens when we examine biological datasets in which biological noise, by necessity absent from the technical replicates that make up control datasets, makes up a significant component of the measured signal?

**Figure 3:**
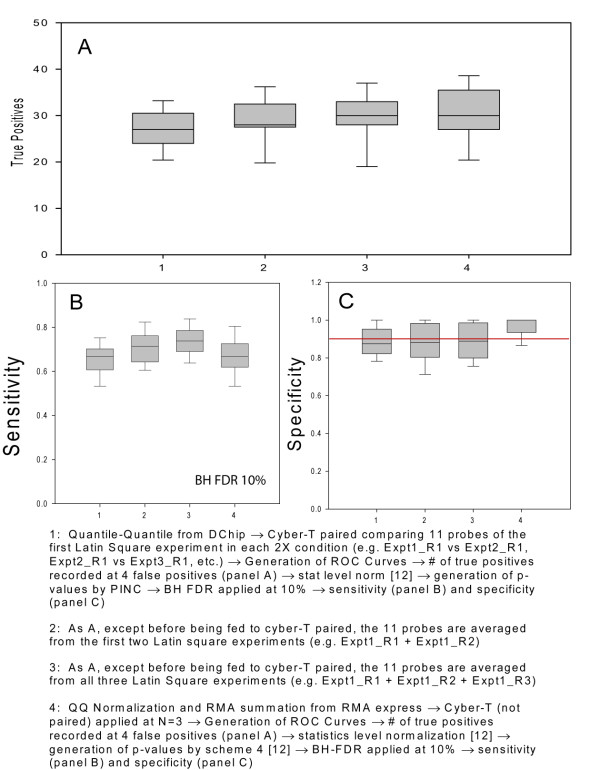
**The effect of sample size on sensitivity and specificity for the 13 Latin Square 2× comparisons**. (A) The number of true positives captured at an arbitrary cutoff of four false positives. Sensitivity (B) and Specificity (C) at a cutoff defined by 10% BH-FDR.

To begin to examine this question we first ask, what are the consequences in the Latin Square experiment of increasing sample size? We applied the PINC algorithm to technical replicates in the Latin Square dataset by analyzing N = 1, N = 2 and N = 3 (conditions 1, 2 and 3 in Figure 3). For N = 2 and N = 3, we determined the average value for each probe and then applied PINC in a pairwise probe to probe comparison similar to when N = 1. By contrast, in most microarray experiments an analysis is performed at the probeset level; that is, an algorithm such as RMA is applied to produce for each probeset on each array a single value and a test statistic is then applied to these values[3]. We therefore included a comparison of PINC to a probeset level analysis, in this case using Cyber-T (not paired as the microarrays in the Latin Square experiment do not have a paired relationship). Condition 4 in Figure 3 shows the results of using quantile quantile normalization and RMA summation[3] to power an analysis with Cyber-T an N = 3.

Figure 3 shows the results of these analyses of different sample sizes on the 13 Latin Square 2× comparisons. Figure 3A shows the number of true positives that can be recovered at an arbitrary cutoff of four false positives (similar to Figure 1C). Figure 3B shows the results of sensitivity and specificity after applying a BH-FDR cutoff of 10% (similar to Figure 2A). We see very similar results no matter if we use 1, 2 or 3 microarrays (conditions 1–3) or use a probeset analysis at N = 3 (condition 4). This confirms the observation of Klebanov and Yakovlev that noise derived from technical replicates is generally low[21] and that PINC can yield results similar to a popular probeset algorithm such as Cyber-T despite the use of only one microarray.

We next applied PINC to a series of biological replicates with varying degrees of biological noise. We chose to analyze an Affymetrix dataset from a cell line study (Accession: NCBI Entrez Geo GDS756) that explored changes in gene expression of SW480, a primary colon cancer cell line [22] and an experiment extracted from human tissue with multiple human donors (Accession: GDS2191) that explores the regulation of the ubiquitin cycle in bipolar disorder [23]. We reasoned that the biological noise in the human tissue dataset would be higher than the biological noise from the cell lines, while the cell lines would in turn have more noise than the technical replicates of the Latin Square experiment [see Additional file 1]. The experiments we chose all met the following criteria; the sample size needed to be at least N = 3, the datasets needed to be a control versus treatment type of design, the datasets needed to be based on the Affymetrix HG-U133A platform and the CEL files publicly available. Within each dataset with N>3, three microarrays for analysis were randomly chosen using a random number selection program .

**Figure 4:**
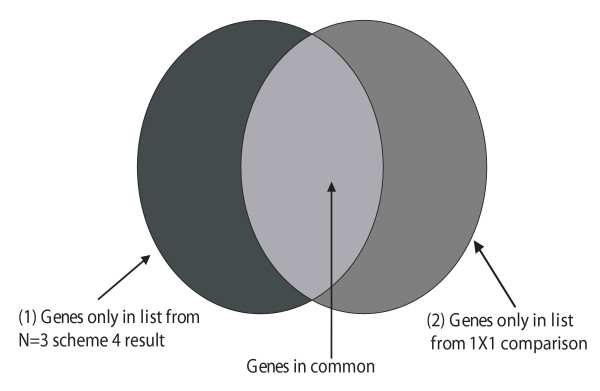
**Venn diagram depicting how genes from each type of analysis are compared in Figure 5**. If biological variability is low, then the majority of genes detected will be common to both methods of analysis.

For each of these datasets, we compared the results obtained when using a probeset analysis with N = 3 with the nine possible analyses comparing individually each of the three chips in each condition. For the probeset analysis, we used "Scheme 4" [14] as described previously, which compares datasets at the probeset level using Cyber-T and then calculates p-values by assuming a single background distribution. A gene list of significant results was determined from "scheme 4" using BH-FDR at 10% FDR. We call these gene results the "Scheme 4 N = 3 probeset results" (condition 1 in Figure 4). Next, using the 3 arrays in each condition, we generated 9 different lists of differentiated genes by performing all 9 possible comparisons using PINC with a single array under 10% BH-FDR (condition 2 in Figure 4). We then compared these 9 results to the "Scheme 4 N = 3 probeset results" to determine how consistent the gene selection process was. Figure 4 depicts a Venn diagram of how these results are interpreted.

**Figure 5:**
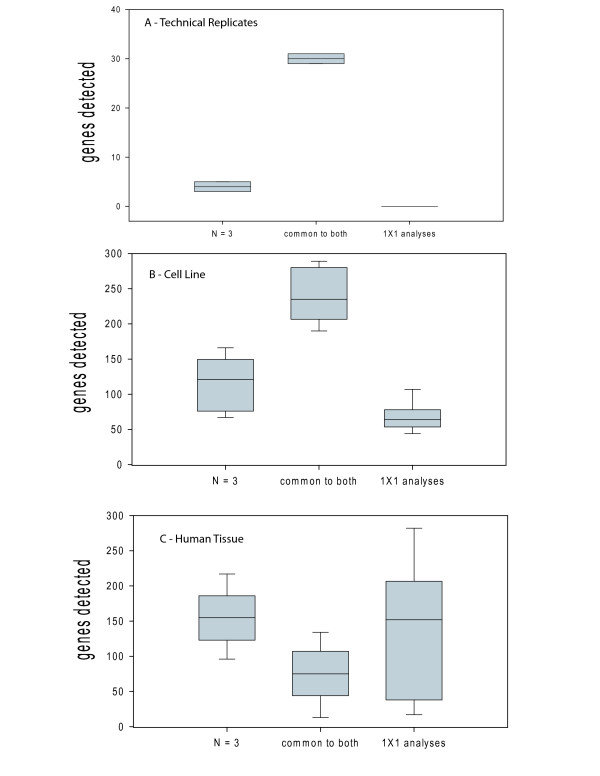
**Comparison of different biological sources using probe-set analytical methods at N = 3 and PINC**. (A) Latin Square dataset – majority of significant genes are common to both methods. (B) Human cell culture dataset – majority of genes still in agreement, although with an increase in variability. (C) Human tissue dataset – very small selection of genes common to both and a large degree of variability in the 1 to 1 comparison group.

Box plots showing the results of these 9 analyses for each dataset are shown in Figure 5. In the Latin Square experiments, genes detected by the 9 different PINC comparisons are in good agreement with the N = 3 gene list (average number of consensus genes found via 1 × 1 comparisons ≈ 88% retained, panel A, Figure 5). As we proceed to the more diverse biological datasets, gene list agreement decreases to 68% and 32% for the cell culture experiment and tissue experiment respectively (panels B and C, Figure 5). For the human tissue experiment, the gene lists generated from the 9 different 1 to 1 comparisons show the highest level of variability (panel C, Figure 5). This is consistent with other tissue microarray experiments we analyzed (data not shown). While this is not a surprise, it does emphasize the danger of analyzing tissue samples via microarray when sample size is low. The extent of variability suggests that when designing a microarray experiment, selection of sample size should reflect the noise of the biological source. These results suggest that a "one-size-fits-all" rule of microarray experimental design (such as always have N = 5) is not always the best use of experimental resources. When biological noise is very low, a single microarray may suffice; when biological noise is high, many microarrays may not capture all of the variability in the system under study.

## Conclusion

Experiments with few numbers of repeats are ineligible for analysis via most published microarray analytical methods. We have shown that when applying analysis at a probe level using PINC, we are able to generate reasonable results on control datasets at N = 1 in each condition. For paired single microarrays, PINC outperforms both canonical statistics and a recently published method[10] while offering conceptually simple statistics and fast run-times. Because the p-values are derived from a distribution estimated from all of the genes on the array, PINC also avoids the large p-values usually associated with low sample size microarray experiments. This allows for the possibility of using a more conservative cut off criteria such as family wise error rate as an alternative to false discovery rate when selecting a p-value cutoff for selecting differentiated genes (Figure 2).

The success of the PINC algorithm in performing accurate inference on the Latin Square dataset at N = 1 suggests that there is little benefit to performing additional technical replicates. This is consistent with previous literature [21] as is our observation that one gets largely similar results whether one uses N = 1, N = 2 or N = 3 in ranking the 2× Latin Square experiments (Figure 3). The ability to analyze single Affymetrix experiments in a statistically rigorous way opens up the possibility of interesting analyses even for experiments in which multiple biological samples were collected. For example, in a cancer study in which cancer tissue is compared against non-cancer tissue from the same patient, we could generate gene lists consisting of genes that are differentially expressed at a given cutoff threshold for every patient in the study. This may yield very different insights than the usual practice of averaging the samples together and performing a single analysis to generate a single gene list. We know that diseases like cancer are very diverse with many different molecular mechanisms presenting similar clinical diagnostics. The ability to evaluate each patient individually in a statistically rigorous way may improve our understanding of the diverse causes of diseases such as cancer and may allow for better use of microarrays in personalized medicine.

## Methods

### PINC Details

PINC harnesses Cyber-T, an algorithm that utilizes a Bayesian probabilistic framework to model log-expression values by averaging the canonical variance with a local background variance estimated from genes with similar intensities on the array [13]. The Cyber-T test can be applied to either paired or un-paired samples. The numerator of the Cyber-T test statistic is the same as in a Standard-T test. The denominator, however, has a correction for the local background variance. For example, an unpaired Standard-T test is calculated by:

$$ttest=\frac{-\left(m_{1}-m_{2}\right)}{\sqrt{\left(\frac{\left(\left(\left(n_{1}-1\right)*SD_{1}^{2}\right)+\left(n_{2}-1\right)*SD_{2}^{2}\right)}{\left(n_{1}+n_{2}-2\right)*\left(\frac{n_{1}+n_{2}}{n_{1}n_{2}}\right)}\right)}}$$

where n_1 _is the number of samples in condition 1, n_2 _is the number of samples in condition 2, m_1 _and m_2 _are the means of samples 1 and 2 and sd_1 _and sd_2 _are the standard deviation for samples 1 and 2. What distinguishes the Cyber-T test from a Standard-T test for unequal sample size is that the standard deviations for samples 1 and 2 are not given by the canonical formula for standard deviation but rather are given by:

$${\text{SD}}_{\text{Cyber-T}}=\sqrt{\frac{{\text{Conf *SD}}_{\text{Window}}^{2}\;+{\text{ (n-1) * SD}}^{\text{2}}}{\text{Conf }+\text{ n -2}}}$$

where n is the sample size (the number of arrays in the condition), SD is the standard deviation as it is usually calculated, SD_Window _is the average of the standard deviation of the 100 genes with the average intensity closest to the average intensity of the gene under consideration and Conf is an adjustable parameter set to 10 by default in the "v1.0beta" of the Cyber-T distribution for R . In a single chip treatment versus control experiment, *n*_1 _and *n*_2 _are equal to the number of probes for a particular gene and *m*_1 _and *m*_2 _are the averages of each group of probes.

For Affymetrix arrays, Cyber-T is typically used following summation of the probes into a single value for each probeset with an algorithm such as RMA[3] (Examples can be seen in [24,25]). As an alternative, PINC applies Cyber-T directly to probes within a probeset to determine gene expression scores. For a GeneChip such as the Affymetrix HG-U133A Array, each probe set contains 11 perfect match probes (we ignore mismatch probes). Thus for a single chip experiment (treatment versus control) PINC compares 11 probes in each position using the paired Cyber-T test (with N = 11).

The Cyber-T test generates a p-value for each gene evaluating the null hypothesis that the gene is identical in both conditions. Because the estimate for the variance of each gene is not independent but is instead dependent upon its neighboring gene scores, the authors of the Cyber-T do not expect the Cyber-T test to follow a simple t-distribution with n1+n2-2 degrees of freedom. Instead, the Cyber-T test assumes that Cyber-T scores will follow a t-distribution with 2 * Conf+n1 +n2 -2 degrees of freedom. We have previously shown that the p-values generated in this way are not very accurate[14].

To determine which genes are differentially expressed, PINC determines p-values by way of "Scheme 4" [14]. Scheme 4 assumes that all the test statistic scores form a single normal distribution and then applies a "Statistical Level Normalization" step which corrects for systematic drift in the t-statistic away from a value of zero [14].

In summary, PINC takes the scores from the paired Cyber-T test at the probe level and uses "Scheme 4" to calculate the p-values rather than using the p-values reported by the Cyber-T software. In this paper, we refer to "Cyber-T" and "Cyber-T paired" as methods that act on the probe level but do not implement Scheme 4 to generate p-values. In our study, PINC is the only algorithm that has p-values generated by Scheme 4.

### FDR and Family-Wise Error Rate algorithms

For the purposes of this analysis, we determined which genes were differentially expressed by either applying a 10% cut off rate via false discovery rates or performed multiple experiment correction via Holm's step down method [20] (p-value cutoff = 0.05).

The Benjamini and Hochberg algorithm (hereafter BH FDR)[18] yields a predicted False Discovery Rate (FDR) for a given gene in a gene list ordered by statistic p-value:

N*p(k)/k

where N is the number of genes in the list and p(k) is the p-value produced by the test statistic under the null hypothesis of no differential expression for gene k in the list. The more conservative Benjamini and Yekutieli FDR algorithm[19] (hereafter BY FDR) relaxes the assumption that the intensities of the genes on the array are independent. The BY FDR for a given gene k in a list of N genes is:

$$\sum_{\text{i}=\text{1}}^{\text{N}}\frac{\text{1}}{\text{i}}\text{ *N*p(k)*/k}$$

### Other statistical tests

At the probe level, we applied the student's Standard-T test (paired and unpaired), and Wilcoxon Rank Sum test. (The idea of applying the Wilcoxon test at the probe level to a single pair of microarrays was first suggested by Affymetrix[2]). The BGX algorithm[10,11] was also applied to the different datasets as a benchmark comparison.

For Cyber-T and BGX we used implementations in R using the Bioconductor package. All other statistical tests were implemented in Java (code available at ). Results for the Wilcoxon nonparametric test were generated from Java source code made publicly available by D. A. Nix .

### Datasets

To assess the effectiveness of PINC, the HG-U133A Latin Square dataset was downloaded from Affymetrix [26]. Two Probe sets with a number of probes other than 11 probes were discarded. For the Latin Square data sets, probesets 209374_s_at, 205397_x_at and 208010_s_at were excluded for all analyses as instructed by the HG-U133A_tag_Latin_Square.xls spreadsheet. We also excluded any probeset not in the spike-in probesets that started with AFFX-. This resulted in 42 true positives and 22,181 true negatives used for assessing effectiveness. The Affymetrix Latin Square dataset was analyzed using N = 1 for all 14 2× fold conditions taking the first experiment (i.e., the CEL file ending in R1) for each condition. CEL files were normalized as indicated using quantile normalization from dCHIP [27] or RMA Express [3,28,29] with background subtraction turned on (except for the BGX algorithm which performs its own normalization).

## Authors' contributions

Both authors contributed equally to this project.

## Supplementary Material

Additional file 1 **Table 1**: Summary of datasets used for biological analysis This table provides the accession numbers for the microarray datasets used in the PINC analysis of biological data. Click here for file
