# Supplementary material for: Determining gene expression on a single pair of microarrays
Source: BMC Bioinformatics. 2008 Nov 21;9:489. doi: 10.1186/1471-2105-9-489 (PMC2605475; doi:10.1186/1471-2105-9-489)
Supplement: Additional file 1 — Table 1: Summary of datasets used for biological analysis This table provides the accession numbers for the microarray datasets used in the PINC analysis of biological data. [file 1471-2105-9-489-S1.doc]

***Table 1: Summary of datasets used in biological data assessment***

| Affymetrix Latin Square  (1) | Human colon cell culture  Accession: GDS756 | Human tissue  Accession: GDS2191 |
| --- | --- | --- |
| **Condition1**  Experiment 4 (N=3) | **Condition1**  GSM21713  GSM21712  GSM21714 | **Condition1**  GSM123256  GSM123261  GSM123263 |
| **Condition2**  Experiment 5 (N=3) | **Condition2**  GSM21715  GSM21716  GSM21718 | **Condition2**  GSM123244  GSM123248  GSM123249 |

(1) Affymetrix Latin Square Data. [http://www.affymetrix.com/support/technical/sample_data/datasets.affx]
